# Integrating Research, Surveillance, and Practice in Environmental Public Health Tracking

**DOI:** 10.1289/ehp.8735

**Published:** 2006-02-27

**Authors:** Amy D. Kyle, John R. Balmes, Patricia A. Buffler, Philip R. Lee

**Affiliations:** 1 School of Public Health, University of California, Berkeley, California, USA; 2 School of Medicine, University of California, San Francisco, California, USA; 3 Institute for Health Policy Studies, School of Medicine, University of California, San Francisco, California, USA

**Keywords:** chronic disease, disease surveillance, environmental exposure, environmental health, environmental health indicators, environmental health policy, environmental monitoring, environmental public health tracking, population health, public health surveillance

## Abstract

The Centers for Disease Control and Prevention in the U.S. Department of Health and Human Services is working with selected state and local health departments, academic centers, and others to develop an environmental public health tracking initiative to improve geographic and temporal surveillance of environmental hazards, exposures, and related health outcomes. The objective is to support policy strategies and interventions for disease prevention by communities and environmental health agencies at the federal, state, and local levels. The first 3 years of the initiative focused on supporting states and cities in developing capacity, information technology infrastructure, and pilot projects to demonstrate electronic linkage of environmental hazard or exposure data and disease data. The next phase requires implementation across states. This transition could provide opportunities to further integrate research, surveillance, and practice through attention to four areas. The first is to develop a shared and transparent knowledge base that draws on environmental health research and substantiates decisions about what to track and the interpretation of results. The second is to identify and address information needs of policy and stakeholder audiences in environmental health. The third is to adopt mechanisms for coordination, decision making, and governance that can incorporate and support the major entities involved. The fourth is to promote disease prevention by systematically identifying and addressing population-level environmental determinants of health and disease.

Environmental conditions and exposures affect health and contribute to chronic disease morbidity and mortality of importance in the United States [Institute of Medicine (IOM) 1988, 2003; Kindig et al. 2003; Lee and Paxman 1997; McGinnis et al. 2002; Rosenstock 2003]. Monitoring of environmental factors is usually directed toward assessing compliance with regulatory mandates [U.S. Environmental Protection Agency (EPA) 2003a] and not focused on assessing health impacts. Surveillance of noncommunicable, environmentally mediated diseases is limited. In 2000, the Pew Environmental Health Commission (PEHC) recommended development of a system to track environmental agents, exposures, and related diseases (PEHC 2000a, 2000b, 2000c).

In 2001, the U.S. Congress appropriated $17.5 million to the Centers for Disease Control and Prevention (CDC) to develop environmental public health tracking (EPHT). The CDC selected competitively and funded 24 state and local health departments and three schools of public health to participate in this initiative (McGeehin et al. 2004). Priority environmental factors initially identified by the CDC include criteria and hazardous air pollutants, drinking water contaminants, persistent pollutants, and lead (CDC 2003a). Diseases identified as priorities are respiratory diseases including asthma, birth defects, cancers, and neurologic disorders (Litt et al. 2004).

Like public health surveillance, EPHT seeks to estimate the magnitude of health problems in populations, detect outbreaks or elevated rates, understand the natural history of diseases, and evaluate control strategies (Teutsch 2000). However, tracking of environmental hazards and exposures and related health outcomes differs from infectious disease surveillance (Ritz et al. 2005). Occupational health surveillance offers a more relevant model. Both occupational health surveillance and EPHT must address chemical agents; long latency of many relevant diseases; multiplicity of exposures; and the need to control economic and institutional behavior, rather than individual actions, to prevent disease. The U.S. Congress identified these concerns (House Committee on Government Operations 1984) and the need for a national reporting system for occupational health (House Committee on Government Operations 1986) in the 1980s. The National Institute for Occupational Safety and Health (NIOSH) provides funding to some states for a Sentinel Event Notification System for Occupational Risks to recognize, report, and prevent certain disorders, including work-related asthma, silicosis, and acute pesticide illness (Baker 1989). This does not provide a comprehensive picture of occupational disease, because geographic areas and disorders included are limited. Even when additional data sources are used, current surveillance does not fully ascertain the extent of workplace-related disease in the United States (Azaroff et al. 2002). This experience suggests potential obstacles.

In EPHT to date, the CDC has emphasized pilot projects to electronically link data and development of specifications for improved systems for the electronic communication and use of data, consistent with broader efforts to modernize public health information systems (IOM 2003; Kufafka et al. 2001; Lumpkin 2001; Yasnoff et al. 2004). The CDC has also funded planning and capacity building, review of data sources, and assessment of indicators (CDC 2002a, 2002b)

In defining a conceptual approach for EPHT, the CDC began with a model including the three elements: hazards, exposures, and diseases (Thacker et al. 1996). This model defines hazard surveillance as “assessment of the occurrence of, distribution of, and secular trends in levels of hazards (toxic chemical agents, physical agents, biomechanical stressors, as well as biological agents) responsible for disease and injury.” It defines exposure surveillance as the “monitoring of individual members of the population for the presence of an environmental agent or its clinically inapparent (i.e., subclinical or preclinical) effects” (Thacker et al. 1996).

The CDC augmented the model by proposing to link data about hazards, exposures, and diseases and to look for possible associations as part of the surveillance system (CDC 2002a, 2002b, 2003a, 2003b). Such data linkages would be accomplished through use of common geographic and temporal identifiers to overlay or combine data over common areas and time frames (CDC 2004a). Most pilot projects funded through EPHT demonstrate data linkages (CDC 2004b). The CDC notes that

A key distinction between EPHT and traditional surveillance is the emphasis on data integration across health, human exposure, and hazard information systems . . . that includes linkage of these data as part of regular surveillance activities . . . . This system will be used to identify potential relationships between exposure and health conditions that either indicate the need for additional research or require intervention to prevent disease, disability and injury.” (McGeehin et al. 2004)

In 2005, the CDC selected four academic centers to participate in the next phase of the EPHT initiative and plans to competitively select state and local health departments for the next phase in 2006. The transition from the first to the second phase provides an opportunity to build on existing work and enhance EPHT by more closely integrating research, surveillance, and practice.

In this article we address four topics relevant to further development of EPHT. The first is to develop a shared and transparent knowledge base that draws on environmental health research and substantiates decisions about what to track and the interpretation of results. The second is to identify and address information needs of policy and stakeholder audiences in environmental health. The third is to adopt mechanisms for coordination, decision making, and governance that can incorporate and support the major entities involved. The fourth is to promote disease prevention by systematically identifying and addressing population-level environmental determinants of health and disease.

## Integrating Research, Surveillance, and Practice

A fundamental tenet of public health is that surveillance should be conducted only when there is “some reasonable expectation of intervention,” i.e., actions to reduce disease or improve health (Teutsch 2000). By integrating knowledge of the environmental factors that contribute to health and disease into research, surveillance, and practice, EPHT can contribute to disease prevention.

### Building a shared base of knowledge to support environmental public health

What to track, how to present and interpret results, and what to recommend about possible interventions are important decisions. Further development of deliberative processes that inform and support such decisions would strengthen EPHT. An initial step would be to begin to define the knowledge base for these decisions. Observations and conclusions supported by research findings and informed by environmental monitoring and public health surveillance might contribute to such a knowledge base.

Developing a knowledge base for EPHT is consistent with the IOM review of the capabilities and needs of the public health system (IOM 2003). The review distinguished between data, information, and knowledge. Data are measurements and facts about individuals, environments, or communities. Information is what is generated when data are placed in context through analysis. Knowledge is what results from an understanding and interpretation of the information. The IOM viewed the CDC as the holder of a “research base that produces the scientific evidence needed to support the regulations in health-related areas that other federal agencies use” (IOM 2003).

Use of a knowledge base could provide a substantiated and transparent basis for the selection of targets for EPHT, methods used, and interpretation of data collected. This could increase accountability by allowing stakeholders to understand rationales for selecting targets and methods. It could also better connect EPHT to the research community.

Models emerging internationally may be useful to consider. In Europe, an environmental health initiative with purposes similar to EPHT emphasizes the relationship between collection/analysis of data and policy making and public access to information [World Health Organization (WHO) European Region 2002, 2003]. The approach envisions a knowledge base about relationships between environmental factors and health outcomes that exists apart from the linkage of data in an electronic information system. Such a knowledge base is seen as the venue for a common understanding of what is known or suspected to be true about how environmental factors are related to health effects or diseases.

Consideration of a knowledge base could affect the methods used for tracking and decisions about whether linking data is the most appropriate approach. It is relevant to consider how thoroughly relationships between environmental hazards or exposures and health outcomes have been investigated and the strength of any association. Some hazards/exposures and diseases have been well studied. In such cases, linkage of surveillance data may not provide new scientific insights unless it offers methodologic innovations or increased power over previous efforts or can contribute to determining the causal nature of an observed association. For cases where associations have been established, it may be more relevant to focus on tracking environmental determinants of disease. It is also relevant to consider whether data or methodologic limitations may cause data linkages to fail to find associations observed in research studies with greater power or ability to control confounders.

In cases where relationships have not been investigated, data linkages may generate hypotheses and lead to important results. In a classic ecologic study, Goldberger found pellagra to be associated with low income and later determined it was related to diet (Mullan 1989). Linkage studies with sufficient power and ability to control for confounding factors may contribute new scientific findings about associations or the causal nature of these relationships. In other cases, targeted research with adequate attention to design issues may be more informative. Issues with the use of ecologic approaches have been reviewed (Mather et al. 2004).

Consideration of the knowledge base is also relevant to interpretation for policy contexts. It would not be appropriate to view data linkage as a necessary prerequisite for interventions in situations where the potential for harm is established. For example, exposure to lead measured in blood has been conclusively associated with diminution of cognitive abilities in children (Needleman et al. 1990). It is not necessary to conduct data linkages to demonstrate this association before taking action to reduce lead exposures. Moreover, to make a case for action, communities may not accept the burden of demonstrating, at a local level, exposure–disease relationships that have been established through research (National Environmental Justice Advisory Committee 2004). Work in California with community organizations suggests that the linking of data may be less important to communities than readily understandable presentations of information (California Policy Research Center 2004).

EPHT may achieve important advances by developing or identifying new data sources or taking steps that increase comparability of data across large areas or populations. Regional efforts to coordinate data collection and analysis across states could improve data and thus lead to new findings. A biomonitoring and tracking collaborative group currently underway in the western states may be a structure that could support such advances. The Public Health Air Surveillance Evaluation—an interagency project to produce geographically resolved predictions of particulate matter and ozone concentrations—provides another example. The project uses models that incorporate both ambient measurements and satellite data to produce estimates of ambient concentrations expected to be more accurate than monitoring data alone (CDC 2004c). The air surveillance project team is exploring uses of these data to assess relationships with acute health outcomes.

Incorporating a knowledge base into EPHT could increase integration between research and surveillance.

### Meeting information needs of policy and stakeholder audiences

The ultimate goal of EPHT is to improve health and reduce disease. This requires actions by a variety of entities with capabilities and responsibilities related to a wide array of environmental factors. Knowledge must be conveyed to many parties, including local, state, and federal health and environmental officials; elected officials; leaders of business, civic, and health organizations; and stakeholders in discussions about environmental health policy.

Many agencies have relevant responsibilities (Lurie 2002). At the federal level, these include the Department of Health and Human Services (including the U.S. Food and Drug Administration, CDC, and NIOSH), U.S. EPA, Department of Agriculture, Department of Housing and Urban Development, Department of Labor (including the Occupational Safety and Health Administration), Department of Transportation, Department of Defense, and Department of Energy (Gostin 2000).

States have principal authority for public health actions as well as jurisdiction over considerable health data. State environmental agencies conduct a great deal of environmental monitoring, often using standard protocols developed with U.S. EPA. Local agencies have varying degrees of authority and capacity for assessments and actions related to environment and health.

Communities also represent an important audience for EPHT, particularly with regard to environmental issues at the local level. Stakeholders can influence policy makers, especially elected officials. Community needs may best be met by blending technical aspects of environmental health sciences with health promotion (Kegler and Miner 2004).

EPHT has engaged a wide variety of partners. An important next step is to look carefully at the information needs of all partners, particularly for policy and stakeholder audiences. Knowledge about environmental health and the significance of the results of tracking activities must be translated into information that is useful and compelling in policy contexts.

The types of information of greatest use to support policies to protect public health cannot be systematically identified from the current research literature (Goodman et al. 2000; Kindig et al. 2003). It is fair to say, overall, that policy audiences seek information in a more distilled and succinct form than researchers do. Policy makers interested in gaining knowledge and information may lack time, expertise, or interest to review and interpret data (Fox et al. 2003). The IOM assessment of the public health system concluded that public health officials must serve as educators for those in policy positions, noting that the public health system must be supported by “political will, i.e., the commitment of elected officials who direct resources and influence based on evidence” (IOM 2003). Another analysis notes that public health policies require “leadership that informs and motivates, economic incentives that encourage and facilitate change, and science that moves the frontiers. Leading change requires facility in brokering partnerships and blending science and community action” (McGinnis et al. 2002).

To achieve this leadership requires approaches that are effective for the intended audiences. Although EPHT is widely viewed as largely synonymous with data linkage, other approaches to representing and explaining impacts of environmental factors on health are likely to be useful.

**Figure 1:**
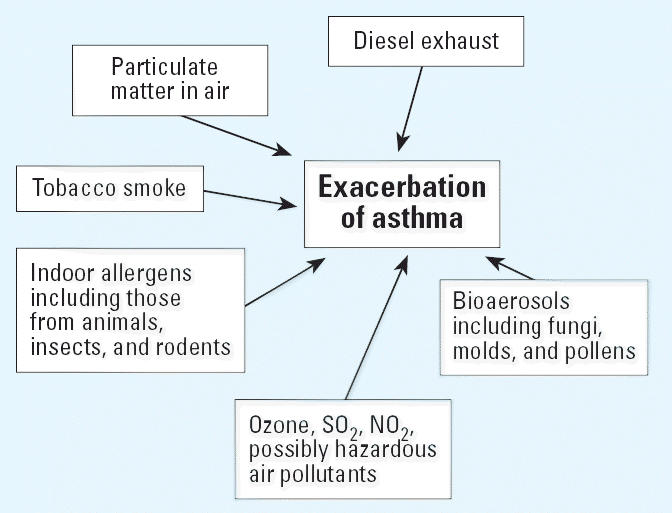
Relationships between environmental factors and health effects: e.g., asthma. Multiple types of environmental factors can contribute to one health outcome.

**Figure 2:**
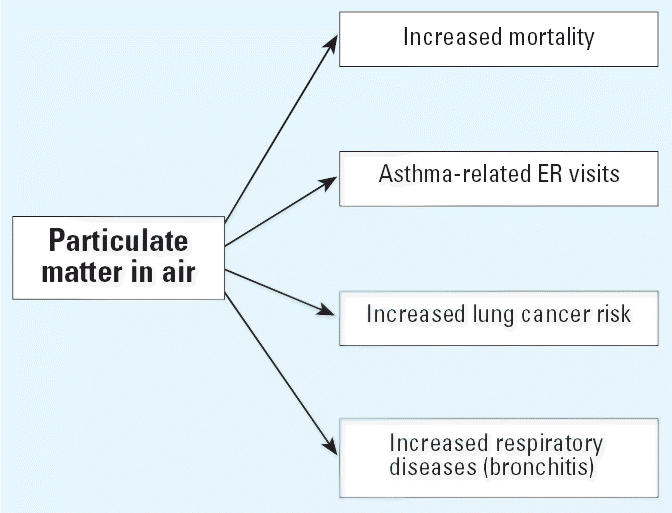
Relationships between diseases and environmental factors: e.g., particulate matter. A single pollutant can contribute to multiple health outcomes.

The technical complexity of relationships between environmental factors and health may require the use of multiple approaches. For example, factors that contribute to disease vary by life stage (Daston et al. 2004), which can be explained conceptually through synthesis of research findings but would be difficult to demonstrate by linkage of data. In another example, multiple relationships between environmental factors and health outcomes often exist, as multiple environmental factors can contribute to a single health outcome. Conversely, a single environmental factor may contribute to many diseases. Figures 1 and 2 show examples related to air pollution and respiratory effects. These relationships can be explained but are not readily illustrated by data linkages. A model that incorporates the idea of multiple exposures and multiple effects is being used increasingly (Briggs 2003). In another example, although it is well known that environmental factors interact with genetic, behavioral, and social factors to affect health, these relationships require interpretation not readily evident by data linkage.

The cost of data linkage can be high in both time and money. The experience of states participating in EPHT is that the effort and resources required to obtain access to data and prepare it for linkage is greater than initially anticipated (unpublished report of the meeting of the western tracking states, February 2005, Berkeley Center for

Environmental Public Health Tracking). Environmental health indicators or measures that summarize technical information in ways relevant to particular audiences may be useful for EPHT. Widely used indicators include the air quality index, reported in many newspapers in the United States, which reflects air quality on a daily basis. Indicators or measures can be scientifically based but portray data about important parameters in ways that are more readily interpretable than the data themselves might be, particularly for policy and stakeholder audiences. Relationships between measures that present data about hazards, exposures, and outcomes may be explained without linking all of the data (Kyle et al. 2006). Such approaches could be included within the conceptual framework for EPHT (Kyle AD, unpublished observations). The review of potential environmental health indicators has been included in the scope of work for EPHT, but the use of such indicators has not yet been integrated into the conceptual approach.

Interest in showing results for certain kinds of governmental actions has increased. One example is the development of goals under the Governmental Performance Results Act of 1993. This has resulted in increased demand for demonstration of the health benefits projected for environmental regulations, such as reductions in cases of disease (such as asthma) associated with reductions in air pollution (U.S. EPA 2003b). Some regard EPHT as a way to document such demonstrations. With sufficient funding of data collection and analyses, in some instances, particularly for acute health effects that vary with short-term changes in environmental conditions, it may be possible to demonstrate such improvements. However, EPHT programs and public health communities need to carefully assess and clearly articulate the circumstances under which such demonstrations can be expected and the resources required to accomplish them.

The next phase of EPHT could better integrate research and surveillance with practice by identifying relevant audiences and developing methods to meet the information needs of these groups in their efforts to promote health and prevent disease.

### Investigating governance structures to support partnership

The EPHT initiative is complex. Many decisions must be made about what to do, how to do it, who controls what, and how to explain and disseminate results. The EPHT network will need to be able to identify needs for decisions, develop and vet proposals, make decisions and commitments, and keep track of what has been done and needs to be done. A model for decision making must incorporate shared expertise, joint priority setting, defined responsibility, and accountability. Approaches to governance, priority setting, and the commitment of resources that facilitate partnership between the environmental and health sectors and among federal, state, and local agencies are sorely needed. Development of a structure of governance to support these needs will be an important challenge for the next phase.

A successful approach would support participation by a wide array of entities. A successful nationwide and sustainable EPHT program requires the long-term participation and stable funding of all states.

Differences in the types of legal authorities available to the public health and environmental protection sectors are relevant. Since the 1970s, legal authority to control environmental factors of health consequence at the federal and state level has been largely vested in environmental agencies. The U.S. EPA plays a lead role and has the authority to formally delegate responsibilities under many statutes to states. There is no analogous authority in the public health sector. The CDC has limited legal authority outside the area of communicable disease. Public health law is widely recognized to be outdated and in need of significant overhaul (Gostin et al. 2003). EPHT program direction has been defined largely in funding agreements. Further definition of a federal role in environmental health among the public health agencies may be worth considering. At a minimum, the implications of these differences need to be further addressed.

Governance structures that can integrate partners engaged in both surveillance and practice and provide a transparent way of making, documenting, and communicating decisions would be valuable.

### Addressing environmental determinants of health and disease for populations

Preventing disease associated with environmental hazards/exposures requires reduction or control of the hazards or exposures. The impact of EPHT would be enhanced with greater emphasis on environmental determinants of disease relevant at the population level. This would be consistent with increased emphasis on determinants of health, which include the physical environment (both natural and built), genetic factors, behavior, and the social environment (Boufford and Lee 2001; IOM 1988, 2003; Lee and Paxman 1997; McGinnis and Foege 1993). Systematic approaches to identify and track known or suspected environmental determinants are an important component of a modern public health system (Lurie 2002). EPHT could provide an opportunity for systematic evaluation of negative and positive determinants stemming from the physical environment and implementation of methods to track them.

Such an approach may require clearer delineation of the various elements that constitute “hazards.” Hazards as defined in EPHT include four conceptually distinct elements: sources of environmental agents, emissions of agents, concentrations of such agents in environmental media (such as lakes or streams), and concentrations in exposure media (such as drinking water). These imply different types of data. The term “hazard” also implies a judgment that these elements pose harm. An approach that can accurately identify, measure, and ultimately influence environmental determinants of health requires more systematic assessment. EPHT represents an opportunity to identify and address such determinants for the environment.

## Conclusion

The EPHT initiative offers an important opportunity to improve data collection and analysis to generate and synthesize knowledge about environmental determinants of population health. The opportunity also exists to increase collaboration and reduce fragmentation between public health and environmental agencies at all levels and to create a technical and organizational foundation for improved environmental public health policy. The goals are ambitious and current resources are insufficient. Further attention to critical needs of the overall program could strengthen it and increase the likelihood of success.
